# Notes and News

**Published:** 1891-09-26

**Authors:** 


### Notes and News.

Collected and Communicated.

Mr. H. S. Gill, M.P., is most kindly giving a children's ward to the Tiverton Infirmary. This is a much needed addition to an excellent institution, for it is a mistake to put adults and children together, especially in a building where space is so valuable as at the Tiverton Infirmary.

Dr. Thorne Thorne, F.R.S., of the Local Government Board, in a paper on "English Isolation Hospitals," draws attention to the great growth in England of the provision by sanitary authorities, and at the cost of the localities, of hospitals for the isolation of first cases of infectious diseases with a view to the prevention of epidemics. At least 400 sanitary authorities now possessed such hospitals, and since 1879, when the author prepared his official report on this subject, about £450,000 had been expended for this purpose. Dr. Thorne says success depends on the proper construction and administration of these hospitals. An unoccupied zone of 40 ft. should surround the hospital buildings, each disease should be treated in a building aerially distinct from any other, the wards should have means of cross-ventilation, and in allotting a space of some 2,000 cubic feet per bed, care should be taken to give at least 12 ft. of wall space to each bed; for it is on a level with the patient's lungs and mouth that it is so essential to secure wholesomeness of air breathed. As a rule, one bed per 1,000 of the population suffices for current permanent requirement, and no district should be content unless they can secure this amount of accommodation.

The Assistant Medical Officers of the Eastern Hospital have addressed a letter to the Metropolitan Asylums Board in which they say: "During the late inquiry, Dr. Collie never allowed blame to fall upon the shoulders of his subordinate officers; but it becomes very difficult to those who in reality, though not perhaps in the eyes of the Local Government Board, share that responsibility to remain silent now that the verdict is published. Reference to the hospital case books will show that in the great majority of cases diets are allotted, *post-mortem* examinations made, medical treatment carried out by ourselves. We cannot, therefore, allow him alone to have the blame for the alleged carelessness and irregularities, which, in so far as they are true, must reflect equally upon us as Dr. Collie's agents. We venture respectfully, but confidently, to assert that had the inquiry into the state of this hospital been in any way complete; that had the good been taken with the bad, and had the bad been thrown into its proper perspective by presenting the great difficulties under which this hospital has laboured, it would have been abundantly proved that we have more reason to be proud than ashamed." This is all very well, but undoubtedly someone is to blame for the disgraceful state of affairs which has existed for so long at the Eastern Hospital. There are not the same complaints about other Metropolitan Board's hospitals; what difficulties does the Eastern Hospital labour under over and above other institutions?

Hope Hospital, which knows no peace, has lately been disturbed because it was bruited abroad that the Matron, being a Roman Catholic, took only Catholics as nurses. As a fact, however, out of 130 persons employed in the hospital there are only eight Catholics, and there is no religious test whatever for applicants.

The foundation stone of the new Poor Hospital for Dundee was laid on September 12th with masonic honours. The principal building will consist of a central block of three storeys, with front elevation facing the south, and on either side two pavilions of two storeys, arranged alternately on a central corridor 323 feet in length. In the central block will be situated apartments for a resident medical officer, accommodation for a staff of nurses, dispensary, waiting-room; and on the uppermost floor, in an isolated position as regards the other wards, confinement rooms and convalescent ward for midwifery cases will be located. The pavilions, four in number, will have on each of the floors one large ward of 30 beds, a small ward of three beds for special cases, a ward, kitchen, scullery, and store closet, together with suitable lavatory accommodation. The architect is Mr. W. Alexander.

The Medical School of Dundee University College has made a further development by the appointment of lecturers in systematic surgery, which supplies a much felt want, and is a decided step in advance, and provides for a complete course for the first two years of study. The clinical lectureships will make a closer union between the School and the Infirmary, and provide a more ample bedside instruction for students while visiting the latter institution. No doubt further wants will be supplied as they become pressing. There is immediate necessity for adequate medical buildings, there being no proper accommodation for the teaching of anatomy and physiology at present. The power now possessed by the College of giving students a complete course for the first two years of their medical curriculum will be of vast advantage to Dundee and district students, as compared with absorption into a body of thousands of fellow-students in Medical Schools like that of Edinburgh. We hope that, as time goes on, the College Council will be put in possession of funds to further extend the teaching powers of the Medical School.

The new Infirmary in connection with the Union workhouse at Cuckfield was opened on the 28th August. This addition to the buildings at Cuckfield is connected with the main part of the building by a light corridor. The corridor leads into a commodious day-room on the lower floor of the new building, which consists of two storeys. On each floor are two large wards, about 36 feet by 20 feet, containing ten beds each. At the end of each ward is a bath-room and lavatories, all fitted with the most approved conveniences. On each floor is also a small ward, containing two beds for special cases, and a nurses' room, these smaller apartments being about 14 feet square. On the upper floor is also provided a large day room for the patients. The wards are well ventilated, and warmed by open fire grates constructed to emit into the wards extra currents of warm air if required. The floors are all of wood blocks. It is proposed to locate the infirm and sick male inmates in the new wing. The old infirmary is being considerably improved and enlarged, and a small isolated infectious hospital has also been built some distance from the main building. The buildings have been designed by Mr. W. Beach, of Haywards Heath, Surveyor to the Board of Guardians. The immediate surroundings of the Infirmary are at present rough, but will eventually be laid out as lawns and gardens.

